# Periorbital Edema Secondary to Positive Airway Pressure Therapy

**DOI:** 10.1155/2015/126501

**Published:** 2015-02-12

**Authors:** Francesco Dandekar, Macario Camacho, Jason Valerio, Chad M. Ruoff

**Affiliations:** ^1^San Diego School of Medicine, University of California, La Jolla, CA, USA; ^2^Department of Psychiatry, Stanford Hospital and Clinics, Stanford, CA, USA; ^3^Sleep Medicine Division, Department of Psychiatry, Stanford Hospital and Clinics, Stanford, CA, USA

## Abstract

Two patients developed bilateral, periorbital edema after initiating positive airway pressure (PAP) therapy with a full face mask. The periorbital edema was more pronounced in the morning and would dissipate throughout the day. This phenomenon seemed to be correlated with the direct pressure of the full face mask, which may have impaired lymphatic and venous drainage. To test this hypothesis, each patient was changed to a nasal pillow interface with subsequent improvement in the periorbital edema.

## 1. Introduction

**Figure 1:**
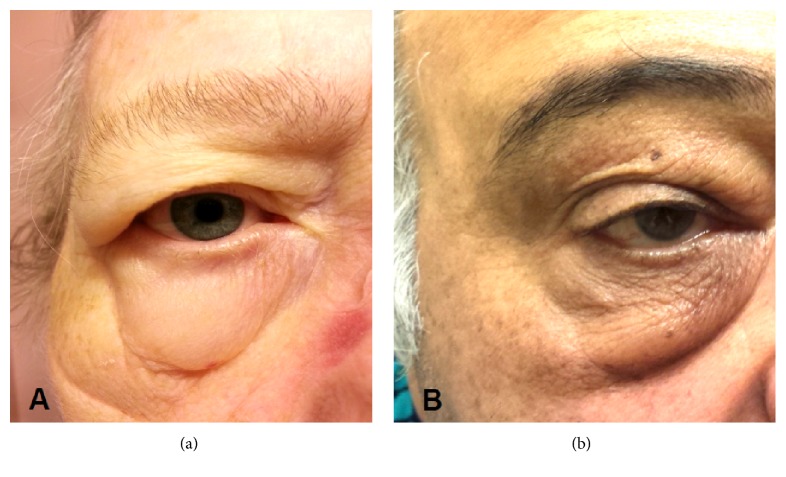
Two patients with periorbital edema after initiating PAP therapy via a full face mask. Periorbital edema developed after starting PAP therapy via a full face mask in patient A in 11 days and patient B in 2 days.

Positive airway pressure (PAP) therapy is commonly utilized as first line treatment for sleep apnea. Complications from PAP therapy are uncommon. Our aim is to describe two patients who developed periorbital eyelid edema (see Figure 1) after initiating PAP therapy for sleep apnea via full face masks.

## 2. Case Presentation

The first patient is a 72-year-old woman with multiple comorbidities, including hypertension, coronary artery disease, fatty liver disease, renal insufficiency, diabetes mellitus, and obesity, who was diagnosed with complex sleep apnea (CSA) and prescribed adaptive servoventilation PAP therapy (maximum pressure of 20 cm of water) via a full face mask. Eleven days after initiating PAP therapy, she developed periorbital eyelid edema, which was most prominent upon awakening and would slowly dissipate across the day (Figure 1(a)). Examination at routine follow-up in the sleep clinic after 3 months of use revealed bilateral eyelid periorbital edema, which was nonerythematous, nontender, and without evidence of infection. The remainder of her exam was unremarkable other than trace bilateral lower extremity edema. In an attempt to identify if high PAP pressure was the cause, her treatment pressure was lowered but no improvement was observed. She then transitioned from a full face mask to a nasal pillow interface with improvement in periorbital edema.

The second patient is a 62-year-old gentleman with multiple comorbidities, including hypertension, hyperlipidemia, and diabetes, who was diagnosed with obstructive sleep apnea (OSA) and prescribed bilevel therapy (maximum pressure of 20 cm of water) via a full facemask. Two days after initiating PAP therapy, he developed periorbital edema, which was most prominent upon awakening and would slowly dissipate across the day (Figure 1(b)). Along with the periorbital edema, he also endorsed periorbital ecchymosis and increased lacrimation. He sought evaluation by a dermatologist and ophthalmologist but no etiology was identified. Examination at routine follow-up in the sleep clinic after 5 months of use revealed bilateral, ballotable, and lower eyelid edema. The remainder of his exam was unremarkable except for mild periorbital ecchymosis. His PAP pressure was reduced without improvement. He then transitioned from a full face mask to a nasal pillow interface with complete resolution of the periorbital edema within seven days.

## 3. Discussion

Reported ocular complaints from PAP therapy include gritty eyes, conjunctivitis, and lacrimation [1]. Our patients denied a history of periorbital edema prior to PAP therapy yet both developed periorbital edema after nighttime PAP. A literature search in PUBMED, Scopus, and Web of Science using MeSH terms “continuous positive airway pressure” and “eye,” with additional searches for “complications,” “orbital,” and “periorbital” in association with “continuous positive airway pressure,” revealed only two other cases of periorbital edema secondary to PAP therapy. In one, the authors attributed the edema to an orbital fracture with sinus communication [2]; in the other, the authors concluded that the direct pressure of the mask on the face caused venous drainage obstruction and lymphostasis [3]. It has been demonstrated that the lower eyelid lymphatics drain into the submandibular glands [4] and the upper eyelid and medial canthus lymphatics drain into the parotid lymph nodes [5]. Therefore, compression of this drainage passageway could lead to edema superior to the contact points of a full face mask. In contrast to the other two reported cases where PAP therapy was simply discontinued, we first tried a decrease in pressure with no improvement. We then changed from a full face mask to a nasal pillow interface with improvement in the periorbital edema; patients reported equivalent usage of both masks. No imaging studies were obtained in either patient since the edema resolved when the alternative masks were used. The reversibility of the edema, as well as the temporal relationship between the switch of the mask type and the resolution of the edema, supports the hypothesis that pressure from a full face mask may impair lymphatic drainage and induce venous congestion.
